# Impaired Collateral Flow in Pial Arterioles of Aged Rats During Ischemic Stroke

**DOI:** 10.1007/s12975-019-00710-1

**Published:** 2019-06-15

**Authors:** Junqiang Ma, Yonglie Ma, Ashfaq Shuaib, Ian R. Winship

**Affiliations:** 1 Neurochemical Research Unit, Department of Psychiatry, Faculty of Medicine and Dentistry, University of Alberta, 12-127 Clinical Sciences Building, Edmonton, AB T6G 2R3 Canada; 2 Neuroscience and Mental Health Institute, University of Alberta, Edmonton, AB Canada; 3 First Affiliated Hospital, Shantou University Medical College, Shantou, Guangdong China; 4 Division of Neurology, Department of Medicine, Faculty of Medicine and Dentistry, University of Alberta, Edmonton, AB Canada

**Keywords:** Ischemia, Aging, Cerebral circulation, Collaterals

## Abstract

Cerebral collateral circulation and age are critical factors in determining outcome from acute ischemic stroke. Aging may lead to rarefaction of cerebral collaterals, and thereby accelerate ischemic injury by reducing penumbral blood flow. Dynamic changes in pial collaterals after onset of cerebral ischemia may vary with age but have not been extensively studied. Here, laser speckle contrast imaging (LSCI) and two-photon laser scanning microscopy (TPLSM) were combined to monitor cerebral pial collaterals between the anterior cerebral artery (ACA) and the middle cerebral artery (MCA) in young adult and aged male Sprague Dawley rats during distal middle cerebral artery occlusion (dMCAo). Histological analysis showed that aged rats had significantly greater volumes of ischemic damage than young rats. LSCI showed that cerebral collateral perfusion declined over time after stroke in aged and young rats, and that this decline was significantly greater in aged rats. TPLSM demonstrated that pial arterioles narrowed faster after dMCAo in aged rats compared to young adult rats. Notably, while arteriole vessel narrowing was comparable 4.5 h after ischemic onset in aged and young adult rats, red blood cell velocity was stable in young adults but declined over time in aged rats. Overall, red blood cell flux through pial arterioles was significantly reduced at all time-points after 90 min post-dMCAo in aged rats relative to young adult rats. Thus, collateral failure is more severe in aged rats with significantly impaired pial collateral dynamics (reduced diameter, red blood cell velocity, and red blood cell flux) relative to young adult rats.

## Introduction

Stroke disproportionately affects the elderly, with risk of stroke doubling every decade after the age of 55 in both sexes [1–3]. Moreover, elderly stroke patients exhibit significantly worse functional recovery and higher mortality compared to younger patients [1–3]. Thus, preclinical studies of the pathophysiology of stroke should be performed in aged animals whenever possible.

After occlusion of a cerebral vessel, tissue surrounding the nonviable infarct core in the penumbra remains viable due to blood flow via the cerebral collateral circulation [4]. Cerebral collaterals are auxiliary vascular pathways that can partially maintain blood flow to ischemic tissue when primary vascular routes are blocked [5–8]. Pial (or leptomeningeal) collaterals are anastomotic connections on the cortical surface that connect distal branches of the anterior cerebral artery (ACA) and posterior cerebral artery (PCA) with distal branches of the middle cerebral artery (MCA) [4, 9]. Clinically, blood flow through the pial collaterals defines the degree of ischemia in the penumbra of cortical infarcts, and thus influences infarct growth, prognosis, and response to therapy [7, 10–12]. Among recent trials of endovascular thrombectomy [13–17], data from the ESCAPE trial that included multiphase CT angiography demonstrated a strong association between robust pial collateral flow before recanalization and favorable outcome after recanalization [13, 18]. The DAWN and DEFUSE3 trials that evaluated patients following late thrombectomy (6 to 24 h after stroke onset) reported significant benefits of delayed endovascular treatment [19, 20]. Notably, patients with “slow-growing infarcts” due to good collateral circulation were selected into DAWN and DEFUSE3 trials [19–21].

Thus, collaterals are a primary predictor of stroke prognosis and response to treatment, but the interactions between collateral dynamics and aging are not known. Rarefaction of cerebral collaterals with aging has been reported in preclinical models [22], and in some cases, collateral therapies have reported differential efficacy based on age [23]. However, age-related changes in the dynamics of collateral flow are not well described, particularly at the level of visually identified pial collaterals. Here, laser speckle contrast imaging (LSCI) and two-photon laser scanning microscopy (TPLSM) were used to evaluate the dynamics of pial collateral circulation in young adult (2 months) and aged (16 months) rats during the first 4.5 h after distal middle cerebral artery occlusion (dMCAo). Retrograde collateral flow was apparent immediately after dMCAo. While collateral vessels narrowed over time in both groups, overall flow was more impaired and failed over time in aged rats relative to adult young rats.

## Materials and Methods

**Fig. 1:**
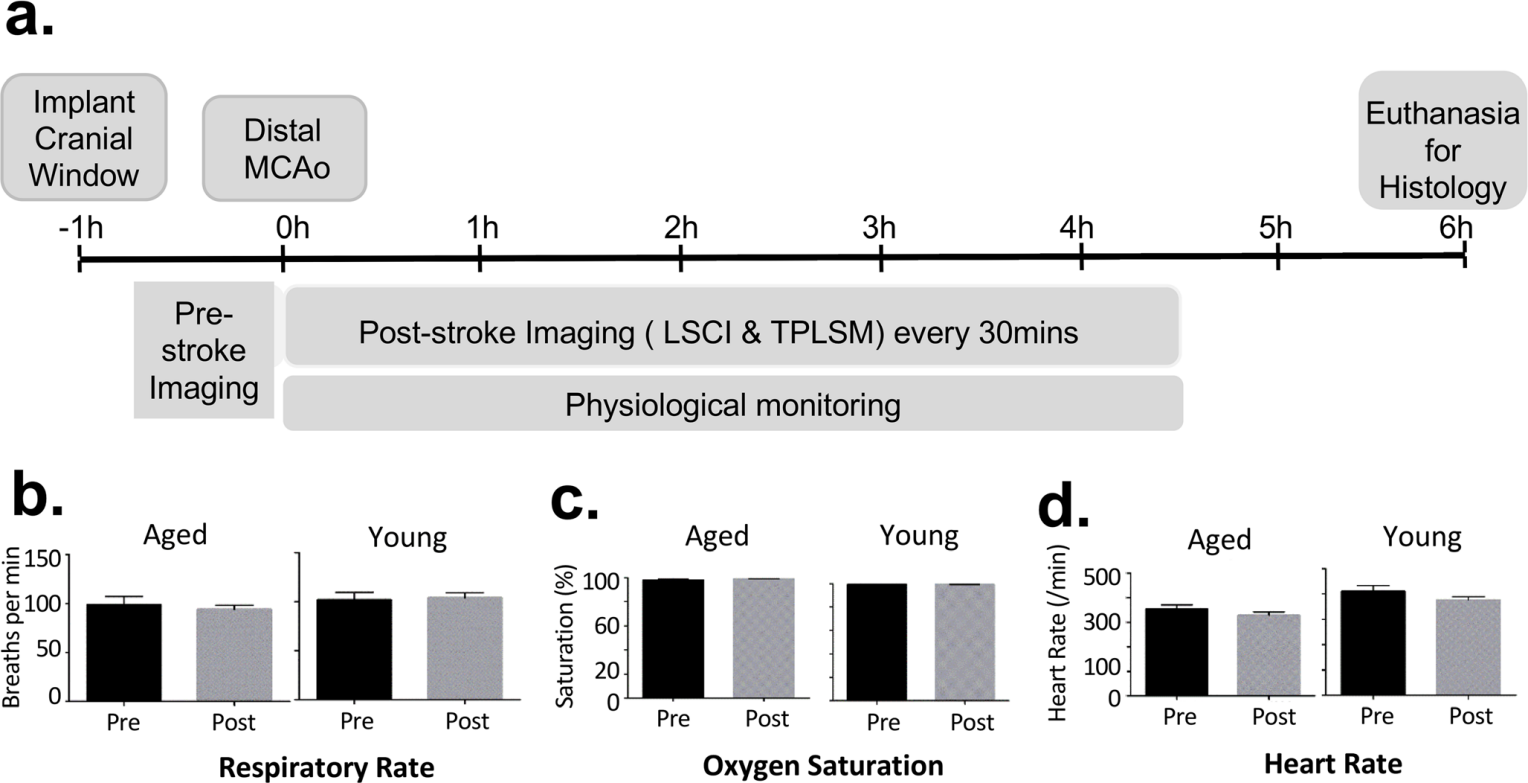
**a** Experimental timeline. **b**–**d** Average of physiological parameters of young and aged rats during the entire poststroke imaging period

Male Sprague–Dawley rats (young group, 2–3 months of age; aged group, 16–18 months of age) were used. Prior to experimental procedures, animals were housed in pairs on a 12-h day/night cycle and had access to food and water ad libitum. Procedures conformed to guidelines established by the Canadian Council on Animal Care and were approved by the Health Sciences Animal Care and Use Committee at the University of Alberta. Procedures and results reporting are consistent with the ARRIVE guidelines [24]. The experimental timeline is illustrated in Fig. 1a. A total of 13 aged rats and 12 young adult rats underwent implantation of an imaging window. Two rats (1 aged, 1 young adult) were excluded due to poor quality cranial windows and image quality (prior to poststroke imaging), and one aged rat died during imaging. Thus, the data set for the aged and young adult groups includes 11 rats each.

### Anesthesia and Monitoring

Light anesthesia was induced using an induction chamber with 4–5% isoflurane (in 70% nitrogen and 30% oxygen) prior to intraperitoneal injections of urethane (1.25 g/kg, divided into four doses delivered at 30-min intervals). Isoflurane was discontinued after the first urethane injection, and rats remained anesthetized until euthanasia. During all surgery and imaging, temperature was maintained at 36.5–37.5 °C with a thermostatically controlled warming pad, and heart rate, oxygen saturation, and breath rate were monitored using a pulse oximeter (MouseOx, STARR Life Sciences).

### Cranial Window

LSCI and TPLSM were performed through cranial windows implanted after craniotomy. A midline incision was made on the scalp to expose the surface of the skull. A 5 × 5 mm section of the skull over the distal regions of the right MCA territory was thinned until translucent using a dental drill (frequently flushing with saline to dissipate heat) and then gently removed. The dura matter was removed, then the cranial window was covered with a thin layer of 1.3% low-melt agarose and sealed with a glass coverslip as previously described [5, 25].

### dMCAo

Cerebral ischemia was induced by bilateral common carotid artery (CCA) ligation in addition with distal MCA ligation [26, 27]. Distal MCA ligation and imaging protocols were performed by different individuals, and surgeons inducing ischemia were blind to the experimental group for each rat. CCAs were accessed through ventral midline cervical incisions and ligated with 4–0 prolene sutures below the carotid bifurcation. A temporal incision was then made and the right temporalis muscle was gently separated from the bone. A burr hole of 1.5 mm in diameter was made through the squamosal bone, the dura was removed, and the cortical MCA was visualized. The exposed distal MCA was isolated with a loose square knot by atraumatic 9–0 prolene suture above the rhinal fissure before stroke. After pre-stroke imaging, the knot was ligated to induce permanent dMCAo.

### LSCI

LSCI measures real-time changes in cerebral blood flow with high spatial and temporal resolution over a wide field of view [28–30]. To collect LSCI data, rats were secured in ear bars on a custom-built stereotaxic plate under a Leica SP5 MP laser scanning microscope. A Thorlabs LDM 785S laser (20 mW, wavelength of 785 nm) was used to illuminate the rat cortex at approximately 30° incidence. Stacks of 101 sequential images (1024 × 1024 pixels) were acquired at 20 Hz (5 ms exposure time) during each imaging session. All processing and analysis of laser speckle images were performed using ImageJ software (NIH) by a blinded experimenter. Maps of speckle contrast were made from the collected images of raw speckling by determining the speckle contrast factor *K* for each pixel in an image. *K* is calculated as the ratio of the standard deviation to the mean intensity (*K* = σ_s_/I) in a small (5 × 5 pixels) region of the speckle image [28–30]. Plots of K show maps of blood flow with darker vessels illustrating faster blood flow velocity [31, 32]. For quantification of penumbral flow, *K* was measured in a contiguous ROI consisting of an 800 × 800 pixel square positioned to include the distal MCA and ACA segments. Because cerebral blood flow (CBF) velocity in the selected region of interest was inversely proportional to the square of speckle contrast value K [33, 34]:

$$ v\propto \frac{1}{k^2} $$

Therefore, 1/K^2^ is also used to illustrate CBF velocity change in LSCI figures [31, 35].

### TPLSM

TPLSM was performed using a Leica SP5 MP TPLSM and Coherent Chameleon Vision II pulse laser tuned to 800 nm. Blood plasma was labeled with fluorescein isothiocyanate–dextran (70,000 MW, Sigma-Aldrich) injected (0.3 mL (5% (*w*/*v*) in saline, 0.2 mL supplements as required) via the tail vein [36, 37]. Z-stacks through the first 0.15 mm of cortical tissue were acquired through the cranial window using a 10 × water dipping objective (Leica HCX APO L10×/0.3 W) and vessel diameter measurements were made from maximum intensity projections of these stacks using ImageJ plug-in (full-width at half-maximum algorithm) [38]. For acquisition of red blood cell (RBC) velocity, line scans were performed in the lumen of arterioles over a length of 50–100 pixels at scan rates of 1200 Hz. While the repeated imaging schedule (30-min intervals) did not allow a comprehensive analysis of blood flow velocity in all vessels within these regions of interest, RBC velocity was via line scans in three identifiable arterioles (> 0.05 mm diameter) per region. RBC velocity was determined from line scan images by calculating the slope of streaks [36, 37]. RBC flux, which provides an overall measure of flow through each vessel, was calculated using the following equation:

$$ \mathrm{Flux}=\left(\pi /8\right)\left({d}^2\right)(v) $$

where *v* is the RBC velocity along the central axis of the vessel, and *d* is the vessel diameter.

### Hematoxylin and Eosin Staining

All rats were euthanized 6 h after induction of the dMCAo. Tissue damage was assessed in digital images of hematoxylin and eosin (H&E)-stained cryosections by a blinded experimenter using ImageJ (NIH) software. Volume of tissues showing early ischemic damage was calculated for each tissue slice using the indirect method [39, 40] to control for tissue distortion due to edema using the following equation:

Volume of ischemic damage % hemisphere = [**Σ(A**_**C**_**-A**_**NI**_**)/ Σ(A**_**C**_)]*100

where A_C_ is the area of the hemisphere contralateral to stroke in a given tissue slice and A_NI_ is the area of the non-injured tissue in the ipsilateral stroke (affected) hemisphere of the same slice.

### Statistical Analysis

Statistical analyses were performed using Graph Pad Prism (GraphPad Software, San Diego, CA, USA). RBC velocity and RBC flux data exhibited a right-skewed distribution. To reduce skewness, a cubed-root transformation was applied. The cubed-root transform was selected as it is a standard transform for right skewness and can be applied to zero values (which occurred in some instances for velocity measurements). Normality was confirmed for all blood flow data sets (i.e., LSCI data in Fig. 3 and TPLSM data in Fig. 5) using the D’Agostino-Pearson normality test. Two-way analysis of variance (ANOVA) with repeated measures was used to compare the time course of aged and young rats on LSCI measures (speckle value, relative blood flow) and TPLSM measures (vessel diameter, RBC velocity, and RBC flux). Post hoc comparisons were performed using the Holm-Sidak  multiple comparisons test. Volumes of ischemic tissue infarct (% of contralateral hemisphere) and physiological parameters (pulse, respiratory rate, and oxygen saturation) were compared using an unpaired Student’s *t* test. Values are expressed as mean ± S.E.M. Sample size was estimated using published and unpublished data that suggested 10 rats were sufficient to detect a 10% difference in vessel diameter using TPLSM (*μ*_1_ = 100, *μ*_2_ = 90, *σ*_s_ = 7.8%, *β* = 0.80, *α* = 0.05).

## Results

**Fig. 2:**
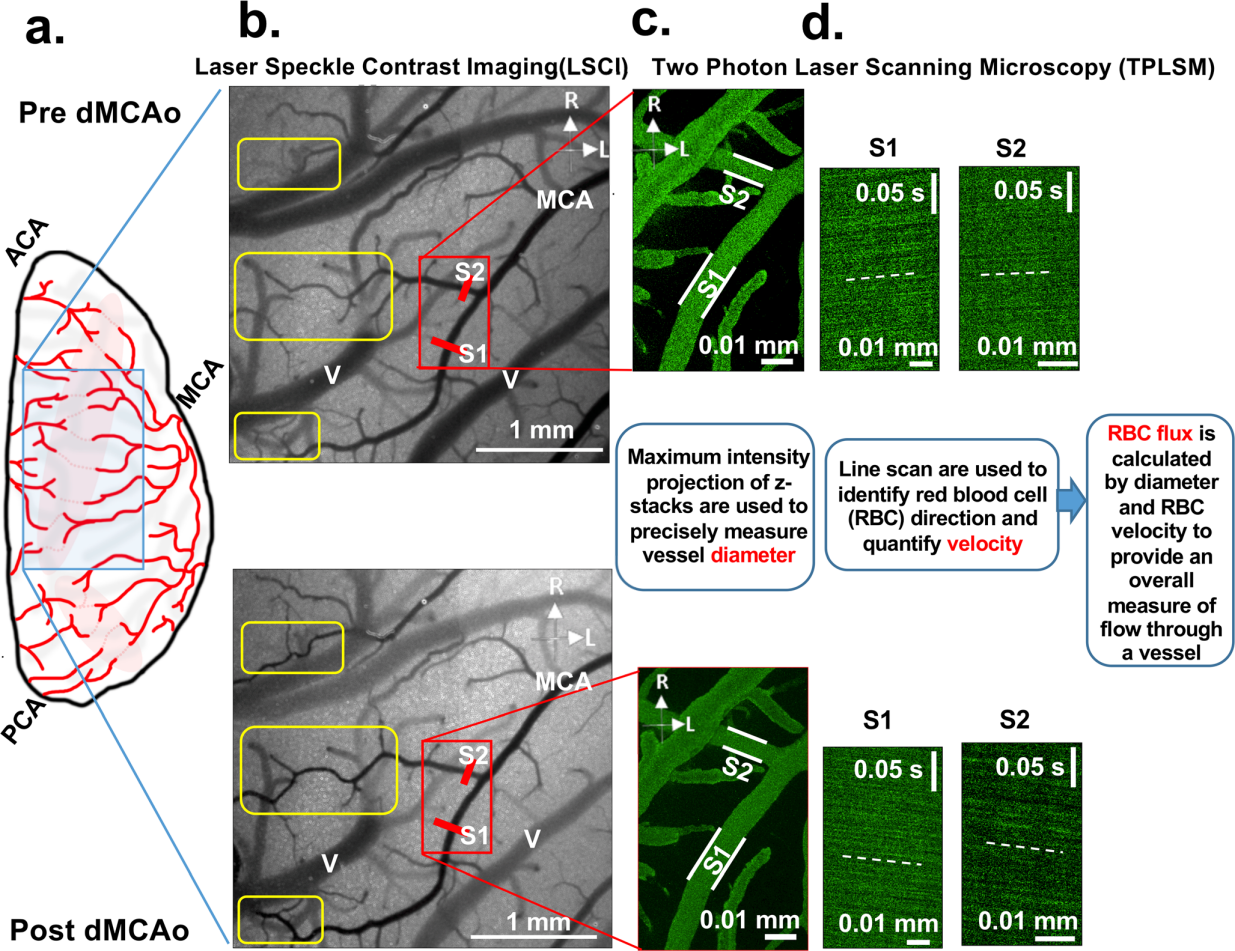
LSCI and TPLSM were used to create high-spatiotemporal–resolution maps of blood flow in pial vessels in the region of ischemia. A cranial window was placed over the cortex at the distal ends of the vascular territories of the ACA and MCA (**a**). Red dotted lines and shading show the approximate locations of ACA-MCA anastomoses. This window placement allows visualization of changes in collateral flow after distal MCAO. **b** LSCI data clearly demonstrate that pial collaterals become patent immediately after ligation of distal MCA (see yellow boxes in **b**), representing retrograde flow from the distal branches of the ACA into the MCA territory. **c**, **d** TPLSM was used to map the angioarchitecture of anastomoses and distal MCA segments. Maximum intensity projections of pial vessels located within a depth of 100–150 μm from the surface of the region demarcated by the red box in (**b**), including distal MCA segments S1 and S2, are shown in (**c**) to illustrate analyses of vessel diameter. **d** Center line RBC velocity was measured in vessel segments measured for diameter, allowing determination of velocity and direction of blood flow. The reversed direction of blood flow in collaterals after MCAO is apparent in both segments (see reversed slope of dashed lines in **d**). Figure 2a modified with permission from Winship et al. [5]. Scale bar, 100 μm

LSCI and TPLSM were used to assess changes pial collateral flow immediately before and for 4.5 h after dMCAO (at intervals of 30 min, Figs. 1a and 2a). Physiological parameters remained stable throughout imaging (Fig. 1b–d). LSCI and TPLSM were used to create high-spatiotemporal resolution maps of blood flow in pial vessels in the region of ischemia, including measures of regional flow (LSCI) as well as pial vessel diameter, RBC velocity, and RBC flux (TPLSM) (Fig. 2) [5].

### LSCI Reveals Reduced Penumbral Blood Flow in Aged Rats Relative to Young Rats

**Fig. 3:**
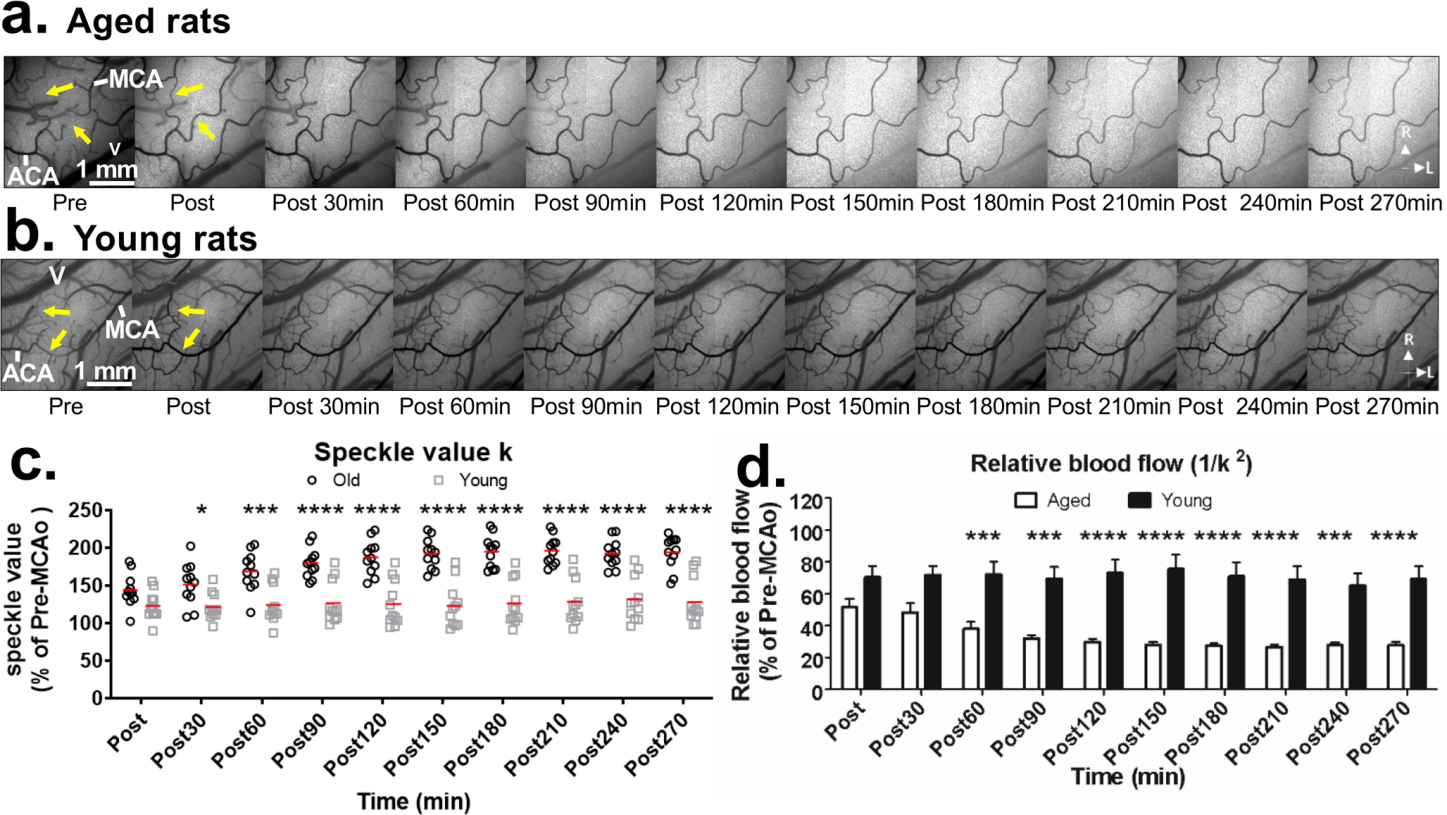
**a**, **b** Representative LSCI-derived image sequences of speckle contrast showing flow on the cortical surface before and after dMCAo. Images showing flow changes over 270 min (4.5 h) post are illustrated for aged (**a**) and young adult rats (**b**). Immediately after dMCAo, robust anastomotic connections between distal segments of the ACA and MCA become visible in both groups (see yellow arrowheads showing absent or low flow in distal ACA-MCA anastomoses before stroke (left-most panel) and enhanced flow after dMCAo in next panel). Pial collaterals were more robust and persistent in young adult rats (*n* = 11) relative to aged rats (*n* = 11). Note the consistent increase in speckle contrast during the imaging period in the aged rats (**a**), which reflects decreasing flow over time, and relatively few visible pial vessels. Contrasting this decreased flow in aged rats, speckle contrast remains relatively stable during imaging after stroke in young adult rats (**b**). Speckle contrast (*k*) and relatively blood flow (1/*k*^2^) for aged and young adult rats are shown in (**c**) and (**d**), respectively. Two-way ANOVA on *k* and 1/*k*^2^ identified significant main effects of age and time × age interactions (all *P* < .0001), and post hoc comparisons identified significantly group differences at all time-points 60 min or more after ischemic onset in both measures. * *P* < .05, ***P < .001, **** *P* < .0001

Figure 3 shows LSCI-derived maps of speckle contrast showing flow changes over 270 min (4.5 h) poststroke for aged and young rats. Immediately after dMCAo, robust anastomotic connections between distal segments of the ACA and MCA were observed in both groups. However, pial collaterals were more robust in young rats (Fig. 3b, note the number of visible vessels following dMCAo in young vs. aged rats), and penumbral blood flow in young rat persisted through the imaging sessions in young rats. In aged rats, penumbral flow decreased during the imaging period (as indicated by a consistent increase in speckle contrast during the imaging period in the aged rats, and relatively few visible pial vessels, Fig. 3a). Speckle contrast normalized to pre-dMCAo values is shown in Fig. 3c. Two-way ANOVA revealed a significant main effect of time and age on speckle contrast, as well as a significant time × age interaction (all *P* < 0.0001). Post hoc comparisons confirmed that speckle contrast was significantly greater in aged rats relative to young rats at all time-points after 30 min post-dMCAo. A more proportional measure of blood flow can be attained by determining the inverse square of the speckle contrast values (Fig. 3d) [31, 35]. While blood flow of young rats remained between 60 and 80% of baseline (pre-dMCAo) during all imaging sessions, flow in aged rats dropped rapidly to less than 40% and remained low throughout imaging. Again, a two-way ANOVA revealed a significant main effect of time and age on speckle contrast, as well as a significant time × age interaction (all *P* < 0. 0001). Post hoc comparisons confirmed significantly reduced flow in aged animals (relative to young animals) at all time-points after 60 min post-dMCAo.

### TPLSM Reveals Dynamic Changes in Pial Arteriole Diameter, RBC Velocity, and Flux After dMCAo

**Fig. 4:**
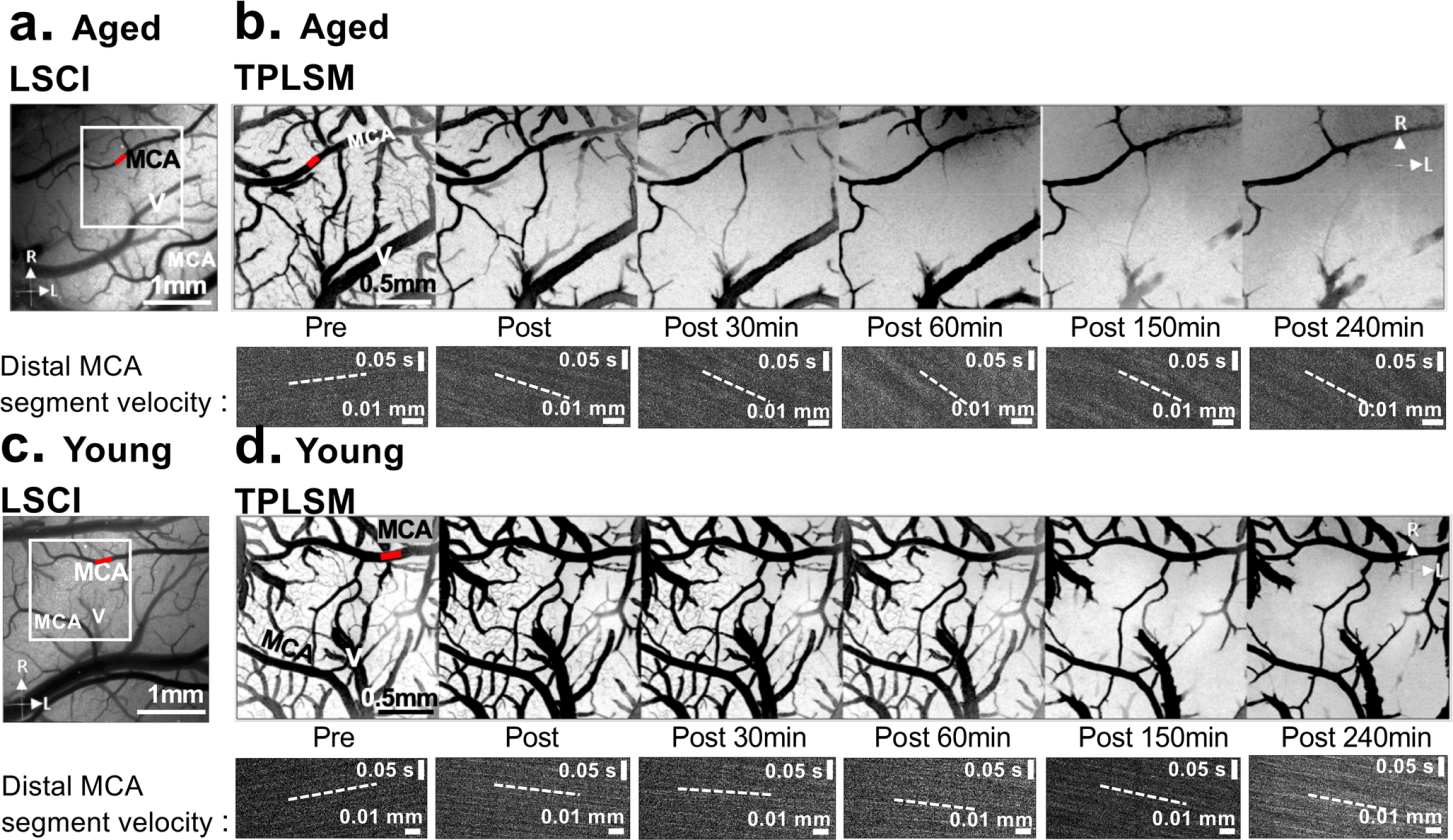
Representative TPLSM data before and after dMCAo. The rectangular box in the LSCI images from an aged rat (**a**) and young adult rat (**c**) demonstrates the location of the representative TPLSM images in (**b**) and (**d**). Scale bar, 1 mm. **b** and **d** show maximum intensity projections from region demarcated in (**a**) and (**c**). TPLSM revealed reduced flow in pial arteriole and diameter over time after dMCAo in both groups, though it was more severe in aged rats. Representative line scans show reduced RBC velocity in the MCA segment highlighted with a red line from the aged rat (**b**) and the young adult rat (**d**). Reversal in the direction of flow is apparent in both groups. Increasing slope in the aged rats shows reduced RBC velocity in this segment, as compared to more stable RBC velocity (and faster, indicated by a lower slope) in the young adult animal. Scale bar, 500 μm. R, rostral; L, lateral

**Fig. 5:**
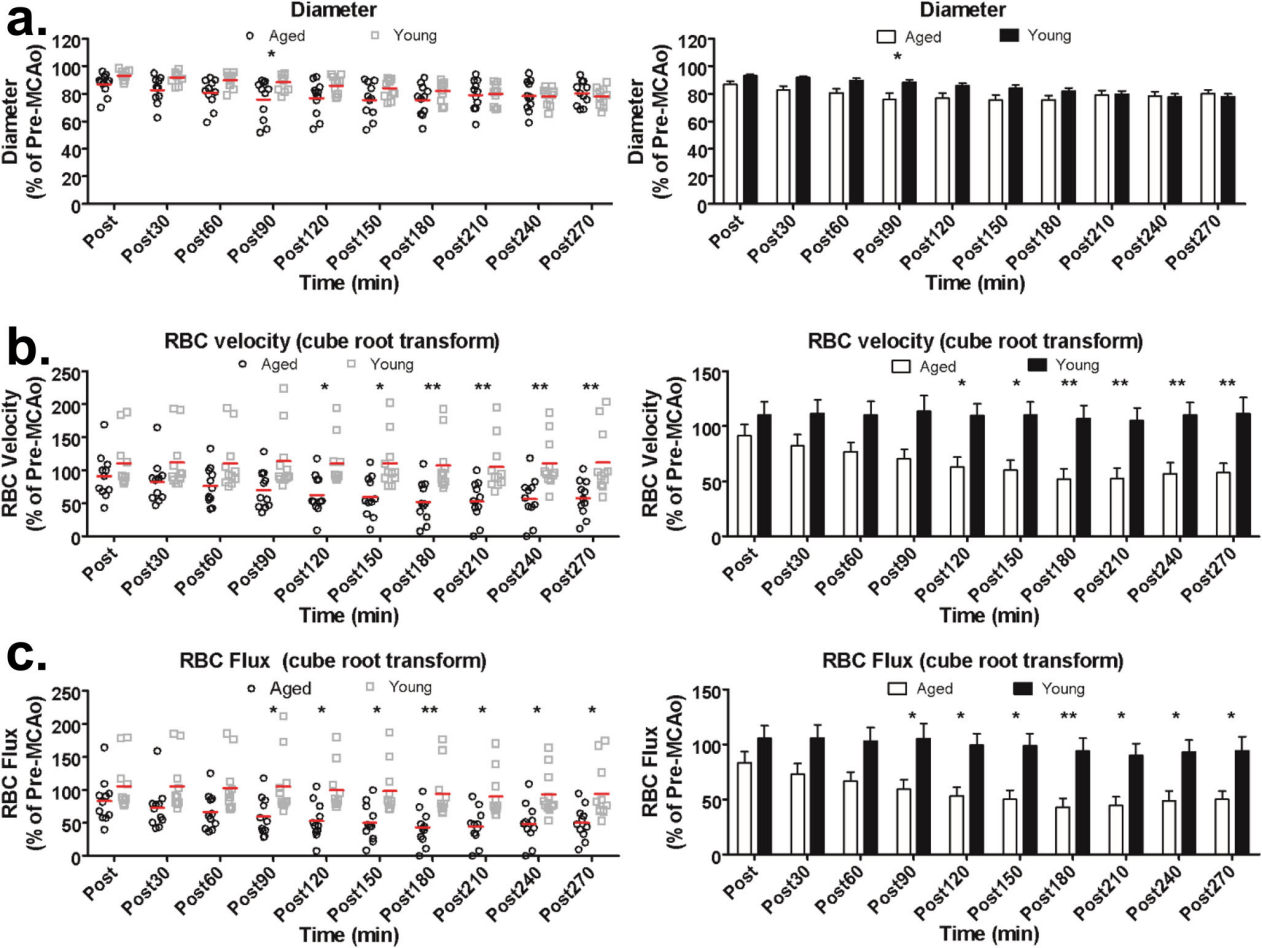
Quantification of the mean diameter (**a**), RBC velocity (**b**), and RBC flux (**c**) in aged rats (*n* = 11) and young rats (*n* = 11) after distal MCAO. **a** Aged rats exhibited a more rapid narrowing of pial arterioles relative to young adult rats, though diameters at the completion of imaging were not different between aged and young rats. Two-way ANOVA confirmed a significant time × age interaction in pial arteriole diameter (*P* < 0.0001). **b** A greater reduction of RBC velocity over time after dMCAo was apparent in aged rats relative to young adult rats, and two-way ANOVA confirmed a significant main effect of time and age (*P* < 0.0001 and *P* = 0.0061, respectively) and a significant age × time interaction (*P* = 0.0002). **c** Mean RBC flux for MCA segments downstream of collateral anastomoses was significantly reduced in aged rats relative to young rats. Two-way ANOVA revealed a significant main effect of time (*P* < 0.0001) and age (*P* = 0.0057), and a significant age × time interaction (*P* = 0.0159). * *P* < .05, ** *P* < .01

TPLSM revealed a reduction in pial arteriole diameter over time after dMCAo in both groups (representative images in Fig. 4, group quantification in Fig. 5). Two-way ANOVA confirmed a significant time × age interaction in pial arteriole diameter (*P* < 0.0001). Notably, aged rats show more rapid “collapse” or narrowing of pial arterioles relative to young rats. Interestingly, diameters at the completion of imaging were not different between experimental groups, and post hoc comparisons only revealed a significant difference in MCA segment diameters at 90 min after dMCAo, suggesting that the dynamics of collateral failure are accelerated in aged rats but the degree of collateral narrowing is comparable. Figure 5b shows mean changes in red blood cell (RBC) velocity relative to baseline (pre-dMCAo). There was a significant main effect of time and age (*P* < 0.0001 and *P* = 0.0061, respectively) and a significant age × time interaction (*P* = 0.0002). Post hoc comparisons confirmed that RBC velocity in pial arerioles downstream of anastomoses was significantly reduced in aged rats relative to young rats at all time-points after 120 min post-dMCAo. Finally, because the oxygen- and nutrient-carrying capacities of a blood vessel are proportional to their RBC flux [41, 42], mean RBC flux for arteriole segments downstream of collateral anastomoses in aged and young rats are shown in Fig. 5c. Analysis of RBC flux between groups revealed a significant main effect of time (*P* < 0.0001) and age (*P* = 0.0057), and a significant age × time interaction (*P* = 0.0159). Post hoc comparisons confirmed significantly reduced RBC flux in aged rats relative to young rats at all time-points after 90 min post-dMCAo.

### Early Ischemic Damage in Aged Rats and Young Rats

**Fig. 6:**
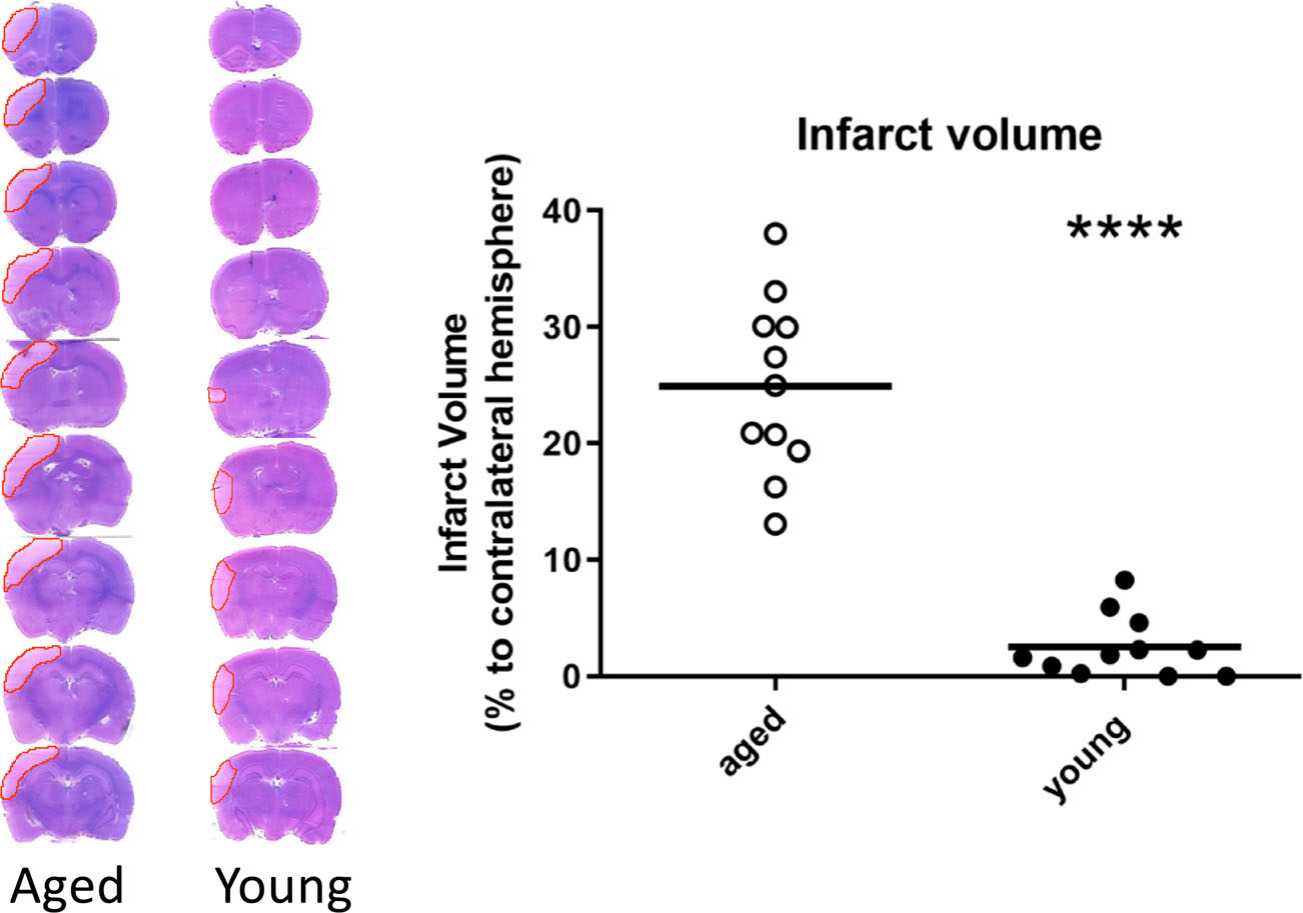
H&E staining was used to visualize early ischemic damage in aged (*n* = 11) and young adult (*n* = 11) rats. A significantly larger volume of early ischemic damage was found in aged rats relative to young rats (*P* < 0.001). Means of the infarct volumes are presented as percentage of their corresponding contralateral sides

Figure 6 shows that a significant larger volume of early ischemic damage was found in aged rats relative to young rats (*P* < 0.001).

## Discussion

Aging is a multifaceted process associated with cellular, metabolic, and structural changes in the brain [43, 44]. Many factors, such as increased oxidative stress, pro-inflammatory cytokine expression, and reduced cell survival, have been considered important factors contributing to increased ischemic brain injury in aged animals [43, 45]. The effects of age on cerebral circulation are additional factors to be considered. Although there is some debate in the field [46–50], most published literature suggest that there is a rarefaction of cerebral arterioles and decrease in capillary density in aged humans, aged nonhuman primates, and different species of aged rodents (such as Wistar, Wistar-Kyoto, spontaneously hypertensive, Brown-Norway, and F344 rats) [51–56]. An age-related decrease in the number of venules and arteriole-to-arteriole anastomoses has also been reported in both Brown-Norway and F344 rats [51]. Such rarefaction would reduce aged animals’ ability to maintain blood flow during ischemia, resulting in increasing risk of neuronal loss in brain regions where vessel rarefaction is prominent [51]. Increasing age is also associated with significantly decreased lumen diameter at the arteriole level and more tortuous cerebral vessels [46, 57, 58]. The net result of these alterations in the cerebral circulation is increased vascular resistance, which leads to impaired tissue perfusion and larger infarcts [59]. A loss of collateral number and diameter and increased tortuosity has been observed in aged mice, resulting in a 6-fold increase in calculated resistance and 3-fold increase in severity of infarct volume after MCAO in 24- versus 3-month-old mice [22, 60]. These studies used postmortem cerebral artery micro-angiography to estimate rarefaction, however, so collateral extent and compensatory flow were not directly verified during ischemia. Notably, age-related biochemical alterations in the peripheral and mesenteric collaterals suggest altered hemodynamics in the days following stroke. Specifically, endothelial nitric oxide synthase (eNOS) signaling appears to be dysfunctional in endothelial cells in mice 3 days after MCAO, as indicated by increased protein nitrosylation and reduced concentration of phosphorylated endothelial nitric oxide synthase [22]. Moreover, expression of vasodilator-stimulated phosphoprotein (VASP) is altered in collateral wall cells [22]. Decreases in phospho-eNOS (necessary for eNOS activation) and phospho-VASP (which undergoes phosphorylation when NO is increased) would impair collateral remodeling during this subacute period, but reduced eNOS could also impair vasodilation and could lead to reduced collateral diameter during acute stroke. This would agree with preclinical and clinical studies in the peripheral and cerebral circulation that suggest impaired vasodilation and vasoreactivity with aging [61–63].

Collateral status at the time of occlusion (i.e., number and diameter) is the strongest independent predictor of final infarct volume and is considered crucial for clinical decision-making in stroke treatment [8, 60, 64–67]. The hemodynamic evolution of the collateral circulation is also important since collaterals are thought to be time limited and can fail over time [25, 26, 68]. The dropout of collaterals during stroke is related to the progression of penumbra to irreversible ischemic infarct and impaired response to treatment [25, 26, 68]. However, the effects of aging on the dynamics of collaterals circulation are not well defined. The data presented here show that pial collaterals are patent immediately after ligation of a distal branch of MCA, with clear retrograde flow to ischemic territories. LSCI showed that cerebral collateral perfusion was impaired after stroke (“collateral failure”) in both aged and young rats, but this decline was more severe in aged rats. TPLSM showed that pial arterioles narrowed to around 80% of pre-stroke diameter at 4.5 h poststroke in both young and aged rats, but this collateral constriction was accelerated in aged rats. More specifically, the narrowing of pial vessels occurred over 90 min poststroke in aged rats, while more gradual narrowing occurred over the full 270 min imaging period in young rats. Notably, RBC velocity remained near baseline values (though the direction of flow was reversed) in young rats, such that overall RBC flux downstream of pial anastomoses was stable over the imaging period. Contrasting this, RBC velocity declined steadily in aged rats after ischemic onset. Thus, while arteriole vessel narrowing reached comparable endpoints, RBC velocity and the overall flux of blood through pial arterioles was significantly reduced at time-points after 120 and 90 min, respectively, after occlusion in aged rats relative to young adult rats. Thus, collateral narrowing occurs more quickly in aged rats than young adult rats, and only young adults compensate for increased vascular resistance with an increase in flow velocity. In addition to changes in the diameter and velocity of flow in pial arterioles, a progressive reduction in perfused vessels on and below the cortical surface was apparent in both aged and young adult rats, but was more severe in aged rats. While potential fading of fluorescence and a slight reduction in the quality of the optics through the cranial window could potentially contribute to reduced density of flowing vessels over time, the consistency of the LSCI and TPLSM images and the progressive reductions in arteriole flux suggest that this reflects an impairment in microvascular perfusion due to failing collateral flow. The more severe reductions in collateral and microvascular flow apparent in aged rats likely account for the significantly greater volumes of ischemic damage relative to young adult rats 6 h after ischemic onset.

Notably, the native pial collateral circulation (number and size) varies greatly among humans and among rodents from different genetic backgrounds, even within a species [60, 69, 70]. Pial collaterals’ formation begins primarily between embryonic day 13.5 and 14.5 and their maturation continues through the first 3 weeks after birth [4, 71, 72]. Gene expression of *Vegfa* and *CLIC4* shapes the development of collateral vessels [4, 71, 73, 74]. However, it is not known how these genetic factors influence collaterals across the lifespan, and if changes in gene expression contribute to accelerated collateral failure. Recent studies of isolated collateral vessels after filament MCAO in rats suggest that the elevation of intracranial pressure (ICP) may be responsible for collateral failure after stroke [75]. While ICP was not monitored in our study, dynamic difference in ICP may occur during acute between aged and young rats and may contribute to accelerated collateral failure observed here. Notably, Beard et al. [75–77] stated that changes in collateral flow poststroke appear to be primarily driven by the pressure drop across the collateral vessel, and were not due to changes in vessel diameter. That is, as ICP increases, cerebral perfusion pressure is reduced and collateral flow declines, providing a possible explanation for collateral failure. In our study, aged rats showed a more rapid narrowing of collateral vessels that was associated with a rapid and sustained decrease in collateral flow. Young adult rats had a slower decline in pial vessel diameter, though diameters at the final endpoint were comparable between groups. However, in young adult rats, blood flow velocity and flux remained relatively stable over time, perhaps implicating a more severe increase in ICP in aged rats that reduces cerebral perfusion pressure as a mechanism of impaired collaterals in the aged. Strategies to reduce ICP may therefore be effective to maintain collateral flow in the aged. Metabolic risk factors, like metabolic syndrome and hyperuricemia, are known to contribute in poor leptomeningeal collateral status of patients with acute ischemic stroke [78]. Menon et al. [78] hypothesized that endothelial dysfunction results from metabolic syndrome and hyperuricemia and leads to pial collateral deterioration [78]. In addition, Faber et al. [22] postulated that endothelial dysfunction could lead to a reduction in the density of cerebral native collaterals in mice. However, the effect of endothelial dysfunction in regulating hemodynamic of pial collaterals poststroke is still unknown, and the degree to which age contributes to this dysfunction remains to be confirmed.

Our finding of rapid failure of collaterals in aged rats may help partially explain worse clinical outcome in elderly relative to young patients. Moreover, our data may help explain results of the completed Safety and efficacy of NeuroFlo Technology in Ischemic Stroke (SENTIS) trial [23]. Notably, the SENTIS trial showed that transient aortic occlusion (TAO) with the NeuroFlo catheter is safe in stroke patients and could improve outcome through augment cerebral blood flow after stroke onset in a subgroup of patients older than 70 years of age [23]. Enhanced efficacy in the elderly may reflect amelioration of ischemia induced by earlier cerebral collateral collapse after ischemic onset in the aged. Accelerated collateral failure as demonstrated here therefore reinforces the importance of early recanalization in the elderly, and suggests that the development of collateral therapeutics to preserve collateral flow might be particularly important for aged patients [23]. Notably, our study did not include reperfusion, and future studies could address the importance of collateral flow prior to recanalization in aged and young rats by incorporating a transient model of MCAo to model stroke with recanalization. Incorporation of approaches to reduce ICP in these studies could highlight the potentially important role of ICP in collateral failure and its potential as a target for collateral therapeutics.
